# The Efficacy and Effectiveness of the Biological Treatment of Pruritus in the Course of Atopic Dermatitis

**DOI:** 10.3390/jcm13061754

**Published:** 2024-03-18

**Authors:** Agnieszka Marta Hołdrowicz, Anna Woźniacka

**Affiliations:** Department of Dermatology and Venereology, Medical University of Lodz, 90-647 Lodz, Poland; anna.wozniacka@umed.lodz.pl

**Keywords:** atopic dermatitis, biological treatment, pruritus

## Abstract

Atopic dermatitis is a heterogenous inflammatory disease with high variety in terms of clinical symptoms and etiopathogenesis, occurring both in pediatric and adult populations. The clinical manifestation of atopic dermatitis varies depending on the age of patients, but all age groups share certain common features, such as a chronic and recurrent course of disease, pruritus, and a co-occurrence of atopic diseases in personal or family medical history. Treating pruritus is a high priority due to its incidence rate in atopic dermatitis and substantial impact on quality of life. In recent years, treatments with biological drugs have increased the range of therapeutic possibilities in atopic dermatitis. The aim of the study is to present the safety profile, efficacy, and effectiveness of various biological treatment methods for the therapy of pruritus in the course of atopic dermatitis.

## 1. Introduction

Atopic dermatitis (AD) is a chronic inflammatory disease estimated to affect up to 20% of children and 10% of adults. In recent years, the incidence rate has been reported to be increasing in both developed and developing countries, probably due to the growing level of urbanization in these areas [1]. It is commonly assumed that the onset of AD occurs mostly in children under the age of five years. However, recent studies suggest that up to 25% of all cases in the adult population first began in adulthood. Furthermore, the occurrence of AD increases the risk of other atopic diseases such as food allergies, allergic rhinitis, and asthma, all of which contribute to the atopic march [2].

Atopic dermatitis is a heterogenous disease entity with high variety in terms of clinical symptoms and etiopathogenesis. Continuous attempts are therefore being made to distinguish and classify various subtypes according to the immunological and genetic profiles of patients and the clinical course of the disease. It is widely expected that finding prognostic and predictive biomarkers will prove beneficial to anticipating the course of the disease in a given patient and applying the best available treatment method [3,4,5]. Possible prognostic biomarkers include serum vascular endothelial growth factor level, modified stratum corneum lipid composition, serum and skin levels of chemokine C-C motif ligand 17 (CCL17), and serum IgE level. Due to the heterogeneity of AD, defining predictive biomarkers is crucial to applying a suitable therapy in the early stage of the disease. The following substances are being examined as promising predictive factors: baseline lesional skin expression of CCL22, chemokine (C-X-C motif) ligand 2 (CXCL2), interleukin-22 (IL-22), serum levels of the dipeptidyl peptidase-4 (DPP4), and periostin [5]. Moreover, in Chinese patients, high levels of CD25/sIL-2Rα, IL-36β, and IL-31 may be related to a good response to dupilumab treatment [3].

The clinical manifestation of atopic dermatitis varies depending on the age of patients, but all age groups share certain common features, such as a chronic and recurrent course of disease, pruritus, and a co-occurrence of atopic diseases in personal or family medical history [2]. Pruritus is not only a source of frustration for patients, but also an important cause of sleep disorders, and significantly impacts the quality of life both of patients and their families [6,7]. It is a fundamental symptom of AD and a primary component of most diagnostic criteria [8]. Both clinical trials and everyday practice use various assessment scales, such as the Numeric Rating Scale (e.g., Peak Pruritus Numerical Rating Scale or PP-NRS), Visual-Analogue Scale (VAS), Verbal Rating Scale (VRS) and the 5-D Itch Scale, to estimate the intensity or occurrence of pruritus [9]. Itch measurement is also a component of Patient-Oriented Eczema Measure (POEM) and Scoring Atopic Dermatitis (SCORAD) scales; both of which assess the overall severity of the disease. It is crucial to define the minimal clinically important improvement in unidimensional scales commonly used for itch intensity evaluation. In an observational study conducted on patients suffering from chronic pruritus, the minimal clinically important difference (MCID) was determined as a reduction of 2 to 3 points in both the NRS and VAS scales [10]. However, more extensive research based on data from four various clinical trials suggests a reduction of at least 4 points on the NRS scale as a clinically meaningful improvement [11].

## 2. Genetics and Epigenetics of Atopic Dermatitis

The pathogenesis of atopic dermatitis is characterized by very high complexity and is influenced by various genetic, epigenetic, immunologic, and environmental factors. A genome-wide association study (GWAS) found 31 locus-containing genes to be related to the pathogenesis of AD. Of these, genes encoding proteins participating in the formation of the epidermal barrier and regulating immunological reactions were believed to be of the greatest significance [12].

One of the most important genetic factors responsible for the dysfunction of the epidermal barrier in the course of AD is a loss-of-function mutation in the filaggrin (FLG) gene. The FLG gene is located on chromosome 1q21in an epidermal differentiation gene complex (EDC) consisting of 27 genes. In the European population, the occurrence of Single-Nucleotide Polymorphism (SNP) variants rs558269137 (2282del4) and rs61816761 (R501X) are most commonly observed [13]. It needs to be pointed out that not all people with a mutation in the filaggrin gene demonstrate symptoms of AD, and similarly, not all patients suffering from this disease carry this mutation [12,14]. However, patients with AD and a recognized mutation in the FLG gene are characterized by an early onset of the disease with a longer and more severe course, palmar hyperlinearity, and a predilection for hands and cheeks. An increased risk of eczema herpeticum, staphylococcal infections, asthma, and allergies is also reported [14].

Patients suffering from atopic dermatitis have presented mutations in genes responsible for encoding other proteins, such as claudins and occludins; these are components of intercellular connections called tight junctions. They have also demonstrated changes in the cystatin A gene, epidermal chymotrypsin and trypsin genes, SPINK-5 gene, epidermal N-methyltransferase gene, and the mast cell chymase gene [12].

Undoubtedly, epigenetic processes such as histone modifications, changes in DNA methylation, and the deregulation of microRNAs also influence the expression of genes associated with the epidermal barrier and immunological reactions. In addition, a number of environmental factors have been implicated in the global increase in AD, i.e., air pollution, tobacco smoke, aeroallergens, infectious pathogens, antibiotics, poor lifestyle and diet, and increasing obesity rate, as well as changes in the gut and skin microbiome, and these may act through alterations in the expression of genes [12,15]. Air pollution has been found to provoke AD and the occurrence of flares in in vivo and in vitro tests and in various long-term epidemiological studies [16]. There is evidence suggesting that in the case of elderly-onset AD, lifetime exposure to air pollutants may be more significant than genetic predisposition [17].

## 3. Pathogenesis of Pruritus in Atopic Dermatitis

Sensory afferent neurons are responsible for the transmission of itch sensations. They are located in the skin and consist of various fiber types, including C-fibers, which can be divided into peptidergic and nonpeptidergic types. Peptidergic neurons are characterized by the expression of various neuropeptides such as calcitonin gene-related peptide (CGRP), substance P, and neurokinins, and by the expression of ion channel transient receptor potential vanilloid 1 (TRPV1), transient receptor potential ankyrin 1 (TRPA1), and GDNF family receptor alpha 3 (GFRα3). Four groups of peptidergic neurons can be distinguished depending on their expression of TRPV1, CGRP (calcitonin gene-related peptide), substance P, and TRPM8 (transient receptor potential cation channel subfamily M member 8).

In contrast, nonpeptidergic neurons generally do not express neuropeptides; however, some also express neurotransmitters and ion channels such as TRPV1, neuromedin B, glutamate, and CGRP. Depending on the expression of the markers in this group, it is possible to distinguish NP1 fibers expressing MAS-related G protein-coupled receptor D (MRGPRD), NP2 fibers with MAS-related GPR member A3 (MRGPRA3), and NP3 fibers with natriuretic peptide B (NPPB). All three types of nonpeptidergic neurons participate in pruritus transmission, but NP1 fibers are mainly triggered by a mechanical stimulus and are also responsible for the transmission of pain.

It should be emphasized that in the course of AD, the amount of sensory nerve fibers is significantly increased in the area of the lesions [18,19]. Moreover, the patients experience a hypersensitivity of the skin—prompting them to itch—in the form of severe and long-lasting pruritus resulting from minor stimuli which would not normally cause any itch sensation [12]. The mechanism of pruritogenesis in AD is very complex. Patients with a damaged epidermal barrier are much more at risk of the penetration of allergens and irritants deep into the skin, together with increased transepidermal water loss and increased exposure to various pathogens. As a result, the presentation of antigens by Langerhans cells and the differentiation of lymphocytes towards Th2 cells take place. Moreover, dry skin results in an enhanced production of nerve elongation factors (NEFs) in keratinocytes and a reduction innerve repulsion factors (NRFs) in the epidermis, which cause the elongation of sensory nerve fibers and their further penetration into the epidermis. This phenomenon explains the skin’s hypersensitivity to pruritogens demonstrated by patients suffering from AD.

Activated Th2 lymphocytes secrete IL-4 and IL-13 [20]. IL-4 has two receptor complexes, viz. type 1 and 2. The type 1 receptor complex consists of IL-4Rα and a gamma chain, while the type 2 complex consists of IL-4Rα and IL-13Rα1. Interleukin 13 binds to IL-13Rα1, which stimulates the formation of a receptor complex with IL-4Rα. IL-13 can also interact with IL-13Rα2, and this complex is most probably formed to dispose of IL-13 [21]. Sensory neurons display both IL-4α and IL-13α1 receptors [12,20,22], but the exact role of these cytokines as pruritogens is not clear and requires further study. However, it is known that IL-4Rα sensitizes the endings of sensory nerves to various pruritogens [22]. It is worth mentioning that IL-13 also sensitizes neurons to bovine adrenal medulla (BAM) 8–22, histamine, and serotonin. Additionally, both cytokines cause the proliferation of neurons in the skin by stimulating fibroblasts to release artemines belonging to NEFs [20]. After binding to receptors, both IL-4 and IL-13 activate the JAK/STAT (Janus kinase/signal transducer and activator of transcription) signaling pathway; therefore, blocking this pathway substantially reduces pruritus intensity in the course of AD [22]. It was proven that the presence of the IL-4Rα R576 polymorphism increases the risk of severe atopic dermatitis [23]. While the level of IL-13 is significantly greater in lesional skin than in non-lesional skin, no such relationship has been found for IL-4. Furthermore, higher levels of IL-13 have been found in the lesions of patients with FLG null mutation than those without the mutation. IL-13 inhibits the expression of filaggrin, loricrin, and involucrin genes [24,25].

Th2 cells also secrete IL-31, which binds to IL-31 receptors on sensory neurons, inducing pruritus. In addition, IL-31RA receptors are also present in epidermal keratinocytes, and their binding with IL-31 results in the secretion of leukotriene B_4_ (LTB4); this further induces itch via LTB4 binding to leukotriene B_4_ type 1 receptor (BLT1) on the fibers of sensory neurons [20]. Moreover, IL-31 stimulates the STAT3-mediated proliferation and viability of small-diameter neurons in the skin. Factors such as scratching, stress, and house dust allergens cause a release of substance P by sensory nerve endings. Substance P also acts as a pruritogen by binding with MrgprA1 on sensory neurons and stimulating the secretion of inter alia NG, tryptase, Tumor Necrosis Factor, histamine, and IL-31 through mast cells; this stimulation is driven by the interaction of NK-1R or MrgprB2/X2 on these cells [20]. It has also been found that *Staphylococcal enterotoxin B* produced by *Staphylococcus aureus* can also stimulate IL-31 expression [12].

Regular mechanical damage to the skin results in the increased expression and secretion of alarmins, including thymic stromal lymphopoietin (TSLP), in epidermal keratinocytes. TSLP causes pruritus both directly, by activating sensory neurons, and indirectly, through stimulating periostin secretion in keratinocytes; thus, TSLP acts as one of the main factors in maintaining the itch–scratch vicious circle [22]. The TSLP heterodimeric receptor, comprising the TSLP receptor (TSLPR) and the IL-7 receptor α-chain (IL-7Rα), is located on C-fibers. After binding to the receptor on the nerve fibers, TSLP activates the TRPA1 ion channel. Interestingly, the activation of transient receptor potential cation channel subfamily V member 4 (TRPV4) by TSLP seems to be related to pruritus occurrence on dry skin [20].

Another alarmin engaged in the development of pruritus, and one that plays a pivotal role in the Th-2 response, is IL-33, a member of the IL-1 family. It interacts with the ST2 (Suppression of Tumorigenicity 2) and IL-1RacP (IL-1R accessory protein) complex, activating macrophages, basophils, mast cells, eosinophils, and type 2 innate lymphoid cells (ILC2). One location of the ST2 receptor is the surface of sensory nerve fibers [20]; however, ST2 exists in both membrane-bound and soluble forms. The membrane-bound form activates the nuclear factor kappa B (NF-kB) signaling pathway after binding with a ligand. IL-33 activates immune system cells, thereby stimulating IL-5, IL-13, and IL-4 cytokine production and mast cell degranulation. It also encourages neutrophil migration [22,26]. IL-5 secretion induces eosinophil chemotaxis, while IL-4 induces the further production of IL-5 and IL-13. It was determined that IL-33 damages the epidermal barrier by inhibiting the expression of protein genes of claudin-1, filaggrin, and antimicrobial peptides (AMPs) [12,22,27].

Another important interleukin in the pathogenesis of atopic dermatitis is IL-22, which is synthesized and released by CD4^+^ T cells, ILC type 3 (ILC3), γδ^+^ T cells, natural killer (NK) cells, CD8^+^ T cells, and dendritic cells (DC). Increased blood and skin IL-22 levels have been reported in patients suffering from atopic dermatitis. Elevated levels of this cytokine were positively correlated with acanthosis, skin barrier defects, and epidermal hyperplasia. It has also been confirmed that IL-22 modulates keratinocyte proliferation and differentiation and stimulates chemokine and antimicrobial peptide production in cells [28]. Moreover, IL-22 inhibits the expression of filaggrin [29]. In addition, IL-22 increases the expression of pruritogenic gastrin-releasing peptide (GRP) on sensory afferent neurons and on immune cells in the skin, and increases the expression of the GRP receptor (GRPR) on keratinocytes [30].

Treating pruritus is a high priority due to its high incidence rate in atopic dermatitis and substantial impact on quality of life. In recent years, treatments with biological drugs, i.e., monoclonal antibodies targeted at cytokines or their receptors, have increased the range of therapeutic possibilities in atopic dermatitis, enabling personalized therapy based on various clinical and laboratory aspects of the disease. The aim of this study is to present the safety profile, efficacy, and effectiveness of various biological treatment methods for the therapy of pruritus in the course of atopic dermatitis, a heterogenous and therapeutically challenging disease.

## 4. Methods

PubMed, Scopus and ClinicalTrials.gov were searched for the phrases “drug chemical name” and “atopic dermatitis”. The final search was conducted on 31 October 2023. Cohort studies, randomized controlled trials, non-randomized studies including real-life observations with at least 20 patients, and long-term open-label studies (min. 2-year-long observation) were selected to perform this non-systematic review. Valuable reviews, case studies, and case series were also included. Multinational clinical trials were preferred when available. The main focus was put on trials with patients over 12 years of age. Only full-text articles were taken into consideration.

The following exclusion criteria were stated: (1) clinical trials with anti-JAK inhibitors, (2) clinical trials of analyzed drugs in other disease entities, (3) articles with no pruritus-related data, (4) studies with identical data to avoid redundancy, (5) articles published before 2010, and (6) non-English publications.

## 5. IL-13- and IL-4-Targeted Therapies

### 5.1. Dupilumab

Dupilumab is a fully human monoclonal antibody aimed at a fragment of IL-4Rα (interleukin-4 receptor subunit α) within the IL-4 and IL-13 receptor complex [31].

Pooled data from four clinical trials, viz. SOLO1, SOLO2, AD ADOL, and CHRONOS, including adult and children over 12 years of age, confirmed that a dupilumab regimen of 300 mg every two weeks (Q2W) reduced both pruritus intensity and incidence rate [32]. Itching was assessed by the Peak Pruritus Numerical Rating Scale and Patient-Oriented Eczema Measure. In the SOLO1 and SOLO2 studies, the participants demonstrated a significantly higher least-squares (LS) mean percent change improvement in daily PP-NRS compared to placebo even by day 2 of therapy [31,32]. In the AD ADOL study, patients below 18 years of age demonstrated an improvement by day 5 [32]. Similarly, in the SOLO 1 and SOLO 2 trials, significantly more patients exhibited an improvement in daily PP-NRS of four points or above on day 4 compared with the placebo group [31,32]. In the AD ADOL study, a similar improvement was noted on day 7 of treatment. Also, dupilumab treatment yielded a significantly higher LS mean change and percentage change in the weekly mean PP-NRS from baseline compared to the placebo group (SOLO1, SOLO2, AD ADOL) and controls (CHRONOS).

Patients receiving dupilumab reported significantly better results regarding pruritus occurrence (POEM scale), and significantly more noted fewer days with itching in the preceding week compared to the control (CHRONOS trial) and placebo groups (SOLO1, SOLO2, and AD ADOL trials) at week 16. The treatment remained efficacious against pruritus for 16 weeks in the SOLO1, SOLO2, and AD ADOL studies and for 52 weeks in the CHRONOS trial [31,32]. In addition, in the SOLO1, SOLO2, and CHRONOS studies, while dupilumab demonstrated pruritus reduction in various skin phenotypes (white, Asian, black, and African American), for the black and African American patients statistically significant outcomes were obtained only for a regimen of 300 mg once a week. This tendency was confirmed by a weight-adjusted analysis. All three racial subgroups yielded a similar safety profile for dupilumab [33].

Although cyclosporine (CsA) is still successfully being used to treat severe atopic dermatitis, it has many adverse reactions and contraindications and should not be applied for longer periods. Combined therapy consisting of dupilumab and topical corticosteroids was also found to be efficacious in patients previously treated with CsA but who had been disqualified from CsA therapy due to intolerance or inadequate response. A statistically significant improvement in weekly average PP-NRS in comparison to controls was observed in the second week of treatment [34].

In a four-year-long observation, i.e., from week 52 to 204 of therapy, further improvements in terms of weekly average Pruritus NRS scores were noticed. In addition, the percentage of patients reporting reduced intensity of pruritus, i.e., by 4 points or more in weekly average Pruritus NRS scores compared to baseline, increased over time: from 66.9% at week 52 to 70.8% at week 204. Only 2.2% of patients were excluded from the trials due to unsatisfactory efficacy, and 4.3% for side effects [35,36]. The data from seven phase 2 and 3 clinical trials confirm that dupilumab therapy is associated with a lower risk of hospitalization due to atopic dermatitis or other reasons among adult patients suffering from moderate-to-severe atopic dermatitis compared to placebo [37].

As clinical practice does not always fully reflect the results of clinical trials, real-life observations are of significant importance. A retrospective study conducted on the data of patients from the Dutch BioDay registry noted a reduction in weekly average Pruritus NRS scores after 16 and 52 weeks of treatment, with 62.1% of patients reporting a reduction of least four points at week 52. During the entire one-year observation, 8.1% of patients withdrew from treatment: 4.3% due to lack of effectiveness and 3.8% as a result of adverse reactions. One-third of patients reported conjunctivitis, the most common side effect resulting in withdrawal [38]. In addition, other prospective and retrospective studies have also noted a significant reduction in pruritus intensity associated with dupilumab therapy; similarly, they found conjunctivitis to be the most common adverse reaction (11–40.9% of patients). Depending on the study, 0.9% to 13.6% of patients resigned from treatment [39,40,41,42].

### 5.2. Tralokinumab

Tralokinumab is a fully human IgG4-class monoclonal antibody that blocks IL-13 from binding to the IL-13Rα1/IL-4Rα receptor complex and the IL-13Rα2 receptor.

Aggregated data from three clinical studies (ECZTRA 1, 2, and 3) including 1976 patients suggest that the antipruritic effect of tralokinumab is visible within the first two weeks of therapy [43]. In the ECZTRA 1 and 2 trials, the group of patients treated with tralokinumab demonstrated itch reduction, and the adjusted mean percentage improvementin the weekly average of worst daily Pruritus NRS from baseline was higher than in the placebo group; it was noted on the second day of treatment. It should be mentioned that patients in the ECZTRA 3 trial were allowed to use local steroids, and this may account for the differences in the outcomes noted in the two groups at the later stage. Further improvements in pruritus intensity were observed up to week 16 of therapy. Additionally, more patients undergoing tralokinumab treatment achieved a reduction in itch intensity of at least four points in comparison to placebo. Along with a reduction in pruritus intensity, patients receiving anti-IL-13 also reported improved sleep quality [43].

The randomized, placebo-controlled ECZTERA 7 clinical trial evaluated the efficacy of tralokinumab in patients with severe atopic dermatitis; all participants had been previously unsuccessfully treated with cyclosporine, either due to inadequate response or intolerance, or were disqualified from such therapy because of contraindications. During a 16-week-long observation, the patients treated with anti-IL-13 were more likely than placebo to report a reduction in pruritus intensity of at least 4 points in the weekly average of worst daily Pruritus NRS compared with baseline. However, no statistically significant differences were found between the two groups. This tendency was maintained for 26 weeks of treatment. It is worth underlining that patients in both groups were allowed to use topical steroids during the whole course of therapy, but the placebo group reported 30% higher total usage than the group undergoing anti-IL-13 treatment [44].

The pooled data from five phase one and two clinical trials up to week 52 of therapy indicate that tralokinumab has a favorable safety profile. Conjunctivitis occurs less often in patients receiving tralokinumab than dupilumab, and there does not seem to be any need to interrupt tralokinumab therapy in the case of vaccinations with inactivated or non-live vaccines [45]. The good safety profile and long-term efficacy of tralokinumab treatment were also confirmed in a two-year-long observation of patients from clinical trials, as only 2.0% of patients resigned from therapy due to unsatisfactory effects and 1.6% terminated treatment as a result of adverse reactions [46].

Real-life observations are short-term and based on very limited groups of patients. In a prospective study of 21 patients from various medical centers in Belgium, a significant improvement in pruritus intensity according to PP-NRS was reported after 16-week therapy. Some patients required simultaneous treatment with cyclosporine or upadacytynib. The best response to the applied tralokinumab therapy was observed in patients who were subjected to immunomodulating medications for the first time, while the worst were noted in patients with prior ineffective anti-JAK treatment. Tralokinumab seems to be less effective than dupilumab in reducing pruritus in the course of atopic dermatitis [47]. However, a retrospective study conducted on 85 patients from 16 different medical centers in Spain found a statistically significant improvement in pruritus intensity and skin lesions after 16 weeks of therapy, together with a reduction inmean PP-NRS score from 8.1 to 3.5 (SD 2.4). The most commonly observed adverse reactions were facial redness and conjunctivitis, both of which occurred in 6% of patients. It should also be noted that over 30% of patients participating in this research had previously been treated with other biological medications or JAK inhibitors [48].

### 5.3. Lebrikizumab

Lebrikizumab is an IgG4-class monoclonal antibody targeting IL-13 and preventing its interaction with the interleukin-4Rα–interleukin-13Rα1 heterodimer signaling complex. However, it does not prevent IL-13 from binding with IL-13Rα1 and IL-13Rα2 receptors, demonstrating its affinity to a different epitope on IL-13 than tralokinumab and cendakimab. After binding to IL-13Rα2, the lebrikizumab and IL-13 complex is degraded in intracellular lysosomes, resulting in further reductions in IL-13 level. In vitro studies also found that lebrikizumab has a particularly strong inhibitory effect against IL-13, demonstrating the highest binding affinity and lowest rate of dissociation among similar inhibitors [25].

Two randomized, placebo-controlled, double-blind clinical studies, ADvocate1 and ADvocate2, comprising 851 patients over 12 years of age, found that significantly more patients treated with lebrikizumab reported a reduction inpruritus intensity by 4 points or more on the Pruritus NRS scale compared to baseline after four weeks of therapy. The most commonly noticed side effect over 16 weeks of treatment was conjunctivitis, affecting 7.4% of patients in ADvocate1 and 7.5% in ADvocate2 [49]. After week 16, patients in both trials who achieved at least EASI 75 or IGA 0/1 (i.e., improvement of at least two points with no rescue medications used during the therapy) were randomly divided into three groups; two groups were treated with 250 mg lebrikizumab every two (Q2W) or four (Q4W) weeks, while the third group received placebo. The observation of patients lasted 36 weeks, i.e., weeks 16 to 52. During this second period, 84.6% of patients receiving lebrikizumab Q2W, 84.7% receiving lebrikizumab Q4W, and 66.3% of the placebo group demonstrated a reduction in pruritus intensity of at least 4 points in the Pruritus NRS scale, which suggests a very good response to the less frequently administered maintenance dose [50].

Pooled data from eight phase two and three clinical trials indicate that the drug has a good safety profile. The most commonly occurring side effect in the group of patients receiving lebrikizumab was conjunctivitis, observed in 8.5% of patients (2.5% in the placebo group) [51].

### 5.4. Cendakimab and Eblasakimab

Two other IL-13 inhibitors, cendakimab and eblasakimab, are currently being evaluated for treating atopic dermatitis. Cendakimab is a humanized monoclonal IgG1-class antibody targeting IL-13 and preventing its binding to IL-13Rα1 and IL-13Rα2. Its efficacy was proven in clinical trials of eosinophilic esophagitis treatment [52]. Cendakimab has recently completed a phase two clinical trial in atopic dermatitis, but the results have not yet been published. Eblasakimab is a monoclonal antibody targeting the IL-13Rα1 fragment, i.e., a component of the IL-13Rα1/IL-4Rα receptor complex, thus inhibiting IL-13 and IL-4 signaling [21,53]. A study based on a small group of patients suggests that the drug may be efficacious in reducing pruritus intensity: after eight weeks of therapy, a significant reduction in PP-NRS in patients receiving eblasakimab was noted in comparison to placebo [53].

### 5.5. CM310

CM310 is a humanized monoclonal antibody against the IL-4Rα receptor that blocks its interaction with IL-4 and IL-13. Its activity was assessed in a multicenter, randomized, double-blind, and placebo-controlled phase IIb trial conducted on 120 patients suffering from moderate-to-severe atopic dermatitis. Patients were divided into two groups receiving 300 mg or 150 mg CM310 subcutaneously every two weeks for 16 weeks, as well as a placebo group. Both treatment groups reported significantly lower pruritus intensity than the placebo group, i.e., a reduction of at least four points in the weekly mean daily PP-NRS compared to baseline. The antipruritic effect of the treatment was noted in the second week of therapy and increased gradually up to week 16. Similar incidences of treatment-emergent adverse events (TEAEs) were noted in all three groups, but patients undergoing CM310 treatment were more susceptible to hyperuricemia and hyperlipidemia [54]. Further clinical trials on the possible usage of CM310 in the therapy of atopic dermatitis are currently being conducted [55].

## 6. IL-31-Targeted Therapies—Nemolizumab

Nemolizumab is a humanized monoclonal antibody targeting the IL-31 receptor *α* (IL-31 RA). In a double-blind, randomized, placebo-controlled phase two clinical trial including 264 patients, nemolizumab was found to have a dose-dependent antipruritic effect in the first week of the treatment; this was reported as a positive least-squares mean percentage change from baseline on the VAS scale. The antipruritic effects of nemolizumab were maintained or even intensified up to week 64 of therapy. The most commonly reported TEAEs were flares of atopic dermatitis, upper respiratory tract infections, and nasopharyngitis [56,57].

Another phase two clinical trials conducted on 226 patients from Europe, North America, and Australia examined the efficacy of various regimens of subcutaneously applied nemolizumab. Patients in one group received 20 mg of nemolizumab on the first day of therapy and then 10 mg every four weeks. Those in the second and third groups received a regimen of 60 mg at the beginning of treatment and a further 30 mg or 90 mg every four weeks during the whole therapy. Patients were allowed to use low-to-medium topical steroids along with the systemic treatment. In the first week of therapy, all nemolizumab regimens displayed a statistically significant reduction in the intensity of pruritus compared to placebo. However, in week 24, the greatest change in PP-NRS score compared to placebo was observed in the patients receiving 30 mg nemolizumab. The incidence of TEAEs was only slightly higher in the treated groups, with the most common adverse reactions being infections such as nasopharyngitis, upper respiratory tract infections, gastroenteritis, and exacerbations of formerly diagnosed asthma [58].

In two multicenter phase III clinical trials (JapicCTI-173740 and JapicCTI-183894) involving 303 adult and child patients over the age of 13 years, it was found that a nemolizumab regimen of 60 mg administered s.c. every four weeks yielded a reduction in pruritus intensity [59]. For the whole duration of the treatment, patients were allowed to use topical steroids and calcineurin inhibitors, as well as antihistamine drugs. After 68 weeks of nemolizumab therapy, a substantial decrease in the VAS scale was reported compared to the beginning of treatment (65.9%). Furthermore, only a minor increase in pruritus intensity was noticed twelve weeks after the last drug administration. Various TEAEs were noted in more than 90% of patients treated with anti-IL-31, but severe TEAEs occurred in less than 5%. The most commonly reported TEAEs were nasopharyngitis and flares of atopic dermatitis; however, it should be mentioned that the AD flares occurred mostly in the first 12 weeks of therapy and decreased with time [59].

## 7. IL-22-Targeted Therapies—Fezakinumab

Fezakinumab is a monoclonal antibody against IL-22. Its efficacy was examined in a randomized, double-blind, placebo-controlled phase IIa clinical trial on 60 adult patients from two medical centers in New York. The patients received fezakinumab intravenously at a starting dose of 600 mg, followed by a regimen of 300 mg every two weeks. Patients suffering from severe atopic dermatitis (SCORAD ≥ 50) demonstrated a statistically significant reduction in SCORAD scoring in comparison to the placebo group after 12 weeks of therapy. No such relationship was reported for all patients treated with anti-IL-22. It is also worth mentioning that no statistically significant difference in the reduction in pruritus intensity was confirmed between patients receiving fezakinumab and the placebo group. However, a decreasing trend in the mean pruritus-related score on the SCORAD scale was observed in patients with baseline pruritus-related values over five up to week 20 of treatment. All analyzed parameters were found to gradually improve until the end of the observation period, i.e., 10 weeks after the administration of the last dose. The two groups reported comparable incidence rates of adverse reactions [60].

Fezakinumab therapy was found to modulate the expression of mRNA of various cytokines in Th22 cells, as well as in Th1, Th2, and Th17 cells. Additionally, among patients receiving fezakinumab, those with high baseline mRNA IL-22 expression demonstrated a more substantial shift in the gene transcription level towards non-lesional skin compared to those undergoing anti-IL-22 treatment with low baseline expression and those receiving placebo with a high level of IL-22 expression. Moreover, patients treated with fezakinumab with a high baseline IL-22 level achieved significant improvements in SCORAD score at both weeks 12 and 20 compared with the beginning of the therapy and with the placebo group. No such correlation was observed for patients receiving anti-IL-22 with a low baseline level of IL-22 [61]. The analyzed data suggest that fezakinumab therapy may be suitable for a limited group of patients with a high baseline level of IL-22, but further long-term trials are needed to confirm these results [60,61].

## 8. TSLP- and TSLPR-Targeted Therapies—Tezepelumab

Tezepelumab is a human monoclonal IgG2 λ antibody targeting TSLP and preventing its binding to the receptor complex [62,63].

A multicenter, randomized, double-blind, and placebo-controlled phase IIa clinical trial examined the effects of tezepelumab treatment on 111 adult patients suffering from medium-to-severe atopic dermatitis. No statistically significant difference was found in the number of patients achieving the primary outcome (reaching EASI 50 response) at week 12 of therapy between the tezepelumab groups and placebo. However, a significant difference in reduction in itch intensity was confirmed based on Pruritus NRS scoring. Furthermore, lower PP-NRS scoring was noted in the tezepelumab group, but was not statistically significant. Patients were allowed to use high-potency topical steroids during the trial. The best results were achieved by patients with high DPP-4 and IgE levels and low periostin level. The rate of occurrence of TEAEs was similar in both analyzed groups. The most frequently observed TEAE in the anti-TSLP group was injection-site erythema [62]. A multiple ascending dose study also reported injection-site reactions in healthy volunteers after the subcutaneous administration of tezepelumab [63].

## 9. IL-33/ST2-Receptor-Targeted Therapies

### 9.1. Etokimab

Etokimab is a humanized monoclonal IgG1-class antibody targeting IL-33. Its safety and pharmacokinetics were tested in a phase IIa clinical trial conducted on 12 adult patients suffering from moderate-to-severe atopic dermatitis. After receiving one 300 mg regimen of etokimab intravenously, all patients achieved an improvement of at least EASI 50 and a statistically significantly lower mean EASI score compared to baseline was noted. Moreover, significant reductions in pruritus intensity and incidence rate were reported in the 5-D Itch Scale [26]. Another randomized, double-blind, placebo-controlled phase IIb clinical study examined the efficacy and safety profile of four etokimab regimens on 302 adult patients with moderate-to-severe atopic dermatitis. None of the groups treated with etokimab reached a primary outcome, determined as percentage change from baseline to week 16 in EASI scoring [64].

### 9.2. Astegolimab

Astegolimab is a human monoclonal antibody targeting the ST2 receptor. The efficacy, safety profile, and pharmacokinetics of astegolimab were examined in a multicenter, randomized, double-blind, placebo-controlled phase II clinical trial on a group of 65 patients suffering from moderate-to-severe atopic dermatitis. Patients received a medication regimen of 490 mg every four weeks. After 16 weeks of therapy, no statistically significant changes were noticed in any analyzed parameters (EASI, IGA, SCORAD, BSA, Pruritus NRS) compared with the placebo group [65].

## 10. IL-36R-Targeted Therapies

### Spesolimab

Spesolimab is a humanized monoclonal IgG-class antibody targeting the IL-36 receptor (IL-36R). It blocks an interaction between IL-36R and the ligand, and thus the activation of the signaling pathway. After excluding from the analysis participants undergoing simultaneous steroid therapy, spesolimab was found to significantly reduce the severity of moderate-to-severe atopic dermatitis (EASI score) at week 16 of treatment compared to baseline in a multicenter, randomized, double-blind, placebo-controlled phase IIa clinical trial. The study did not evaluate the efficacy of treatment in terms of pruritus intensity [66]. Due to unsatisfactory results, spesolimab therapy is no longer undergoing clinical trials for atopic dermatitis [55].

## 11. IL-12/23-Targeted Therapies

### 11.1. Ustekinumab

Ustekinumab is a fully human IgG1-class monoclonal antibody targeting subunit p40, a component of interleukins IL-12 and IL-23. Case reports and case series suggest that ustekinumab may be effective in the treatment of pruritus in the course of atopic dermatitis [67,68,69,70]. However, no satisfactory response was confirmed in phase II placebo-controlled clinical trials [71,72] or more extensive real-life observations [73]. No further clinical studies on this matter are currently being conducted [55].

### 11.2. Risankizumab

Risankizumab is a monoclonal antibody targeting subunit p19 of the interleukin IL-23. The efficacy and safety profile of risankizumab in the treatment of atopic dermatitis was evaluated in a multicenter, randomized, double-blind, placebo-controlled phase II clinical study conducted on a group of 172 adult and child patients aged over 14 years. After 16 weeks of therapy, none of the treatment groups were found to have significantly higher numbers of participants demonstrating EASI75 improvement compared to placebo. In addition, only 13.6% of patients receiving 150 mg risankizumab, and 15.2% receiving 300 mg, achieved a pruritus reduction of at least four points on the Worst Pruritus NRS scale. Due to the unsatisfactory outcomes, the trial was terminated after 16 weeks [74].

## 12. IL-17-Targeted Therapies

### 12.1. Secukinumab

Secukinumab is a human monoclonal antibody against IL-17A preventing its binding to the receptor. A randomized, double-blind, placebo-controlled phase II clinical trial assessed the efficacy of secukinumab therapy on 41 patients with moderate-to-severe atopic dermatitis. The treatment group received 300 mg secukinumab weekly for four weeks followed by 300 mg every four weeks. At week 16 of therapy, no molecularly, histopathologically, or clinically significant response to the treatment was noticed [75].

### 12.2. MOR106

MOR106 is a humanized monoclonal IgG1-class antibody targeting IL-17C. Four first- and second-phase clinical trials indicate that it lacks efficacy in atopic dermatitis therapy with regard to skin changes and pruritus [76].

## 13. OX40- and OX40L-Targeted Therapies

OX40 is a co-stimulator belonging to the TNF superfamily. It is expressed mostly on effector and regulatory T cells, while its ligand OX40L is expressed on activated antigen-presenting cells, such as endothelial cells, B cells, dendritic cells, ILCs, NK cells, T cells, mast cells, and macrophages. The interaction of OX40 with OX40L causes the expansion of effector T cells Th1, Th2, Th17, and Th22 by inhibiting their apoptosis and stimulating their cytokine production. It also encourages the development of T helper memory cells. IL-25 and TSLP increase the expression of OX40L on dendritic cells, inducing the differentiation of OX40-positive T cells. Interleukin 33 stimulates the expression of OX40L on dendritic cells and type two innate lymphoid cells [77,78].

### 13.1. Rocatinlimab

Rocatinlimab is a human monoclonal IgG1 antibody targeting the OX40 receptor on activated T cells, therefore reducing their activity and also the amount of T cells in the mechanism of antibody-dependent cell-mediated cytotoxicity.

The efficacy and safety profile of four different rocatinlimab regimens were examined in a multicenter, randomized, double-blind phase IIb clinical trial involving 273 patients with moderate-to-severe atopic dermatitis. Depending on the group, the patients received rocatinlimab every four weeks (150 mg or 600 mg) or every two weeks (300 mg or 600 mg) for 18 weeks. No patients were receiving topical or systemic immunosuppressive therapies at the time. At week 16, all treatment groups demonstrated a higher percentage of patients with a reduction in pruritus of at least four points (Pruritus NRS scale) compared to the placebo group. In addition, the patients treated with rocatinlimab exhibited a higher mean percentage change from baseline in Pruritus NRS score in comparison to the placebo group. Further decreases in pruritus were observed up to week 36 of therapy and gradually receded after the termination of treatment. The safety profile was similar in all groups, with the most commonly reported adverse reactions being pyrexia, chills, headaches, aphthous ulcer, and nausea [29]. Currently, recruitment is underway for seven various phase III clinical trials on rocatinlimab in adults and adolescents suffering from atopic dermatitis [55].

### 13.2. Amlitelimab

Amlitelimab is a human IgG4-class monoclonal antibody targeting OX40L. It binds to the ligand, blocking its reaction with the OX40 receptor, but causes no reduction in OX40^+^ T cells [77,79].

Amlitelimab treatment for atopic dermatitis was evaluated in a double-blind, randomized, placebo-controlled phase IIa clinical study. Eighty-nine adult patients with moderate-to-severe atopic dermatitis were divided into three groups. Two groups received intravenous amlitelimab, one with a starting dose of 200 mg followed by 100 mg Q4W for a period of 12 weeks, and the other with a starting dose of 500 mg followed by 250 mg Q4W, also for 12 weeks. After 16 weeks of therapy, an EASI75 response was observed in 59.3% of patients treated with the lower regimen and 51.9% of those receiving the higher dose. In addition, 57.9% of patients receiving the lower dose demonstrated a reduction in pruritus intensity of at least four points (Pruritus NRS scale), compared to 62.5% of patients treated with the higher dose. During the 36-week study, TEAEs were reported by 35% of patients receiving the lower dose, and 17% with the higher dose. Additionally, a significant decrease in IL-22 serum level was reported after 16 weeks. No such correlation was observed in the placebo group [77]. Four further clinical studies on amlitelimab in atopic dermatitis are currently registered [55].

## 14. IL-5/IL-5R-Targeted Therapies

### 14.1. Benralizumab

Benralizumab is a humanized monoclonal antibody targeting the ⍺-chain of the IL-5 receptor (IL-5R) expressed on eosinophils and basophils. After binding to its receptor, benralizumab induces cell apoptosis via an antibody-dependent cytotoxic reaction, resulting in an almost complete deletion of eosinophils from the peripheral blood, lungs, and respiratory tract, and a significant reduction in eosinophil production in the bone marrow [80].

One study reports a case of remission in atopic dermatitis during treatment with benralizumab due to uncontrolled asthma [80]. Elsewhere, a randomized, double-blind, placebo-controlled phase II clinical trial (HILLIER) examined the efficacy of benralizumab in a group of 194 patients aged over 12 years suffering from moderate-to-severe atopic dermatitis; after 16 weeks of treatment, no difference was found between the placebo and study groups with regard to the number of patients achieving primary and secondary outcomes. However, a substantial decrease in mean eosinophil count in peripheral blood was reported in the treatment group compared with placebo. Due to these unsatisfactory results, the trial was terminated [81].

### 14.2. Mepolizumab

Mepolizumab is a humanized monoclonal IgG1-class antibody that binds to IL-5 and neutralizes it. A multicenter, randomized, double-blind, placebo-controlled phase II clinical study examined the effect of 100 mg of mepolizumab every four weeks on moderate-to-severe atopic dermatitis. The study included a treatment group of 17 adults and a control group of another 17. The research was terminated due to a lack of satisfactory clinical results [82].

## 15. Anti-IgEAntibodies

### 15.1. Omalizumab

Omalizumab is a humanized monoclonal IgG1-class antibody targeting the Cε3 IgE fragment. It binds to free IgE, preventing its interaction with receptors, which reduces the expression of FcεRI on cells.

The efficacy of omalizumab in the treatment of atopic dermatitis is questionable [83,84]; however, some patients exhibited improvements in skin lesions and pruritus [85,86,87]. Omalizumab was found to demonstrate improvements in skin lesions and quality of life in a randomized, placebo-controlled clinical trial including 62 child patients with AD (4–19 years old) [86].

### 15.2. Ligelizumab

Ligelizumab is a humanized, monoclonal IgG1-class antibody targeting IgE; its affinity to human IgE is several dozen times greater than that of omalizumab. A multicenter, randomized, double-blind, placebo-controlled study found ligelizumab not to be significantly more efficacious than placebo in treating skin changes and reducing pruritus intensity [88].

A summary of biological drugs used in the treatment of atopic dermatitis in the therapy of pruritus is shown in Table 1.

## 16. Conclusions

Many new and promising biological treatments for atopic dermatitis have recently been developed and tested. Some demonstrate high efficacy and a favorable safety profile in clinical trials. The U.S. Food and Drug Administration (FDA) approved dupilumab (2017) and tralokinumab (2021) for use in treating atopic dermatitis, and both medications have demonstrated high effectiveness in real-life observations. However, further clinical trials, head-to-head studies, and real-life observations are needed to develop a specific treatment targeting pruritus.

## Figures and Tables

**Table 1:** Biological drugs in the treatment of pruritus in atopic dermatitis.

Biological Drug	Drug Target	Clinical Trials		Antipruritic Effect	
		Name/Type (Number)	Patients	Clinical Trials	Real-Life
Dupilumab	fragment of IL-4Rα (interleukin-4 receptor subunit α) within the IL-4 and IL-13 receptor complex	*SOLO1* (NCT02277743), *SOLO2* (NCT02277769), *AD ADOL* (NCT03054428), *CHRONOS* (NCT02260986) [31,32], *LIBERTY AD CAFÉ* (NCT03054428) [34]	*SOLO1*, *SOLO2*, *CHRONOS*, *LIBERTY AD* *CAFÉ*: adults *AD ADOL*: adolescents > 12 years of age	significant reduction of pruritus intensity *SOLO1* and *SOLO2* –significantly higher least-squares mean percent change improvement in daily PP-NRS compared to placebo by day 2 of therapy –significantly more patients exhibited an improvement in daily PP-NRS (≥4 points) on day 4 compared with placebo *AD ADOL* –significantly higher least-squares mean percent change improvement in daily PP-NRS compared to placebo by day 5 –significantly more patients exhibited an improvement in daily PP-NRS (≥4 points) on day 7 compared with placebo *LIBERTY AD CAFÉ* –statistically significant improvement in weekly average PP-NRS in comparison to controls in the second week of treatment	Significant reduction of pruritus intensity [39,40,41,42]
Tralokinumab	IL-13	*ECZTRA 1* (NCT03131648), *ECZTRA 2* (NCT03160885), *ECZTRA 3* (NCT03363854) [43], *ECZTERA 7* (NCT03761537) [44]	adults	significant reduction of pruritus intensity *ECZTRA 1, 2* and *3* –antipruritic effect visible within the first two weeks of therapy (aggregated data including 1976 patients) *ECZTRA 1* and *2* –confirmed itch reduction, the adjusted mean percentage improvement in the weekly average of worst daily Pruritus NRS from baseline higher than in placebo; noted on the second day of treatment *ECZTERA 7* –patients more likely than placebo to report a reduction of pruritus intensity of ≥4 points in weekly average of worst daily Pruritus NRS compared with baseline	significant reduction of pruritus intensity [47,48]
Lebrikizumab	IL-13	*ADvocate 1* (NCT04146363), *ADvocate 2* (NCT04178967) [50,51]	over 12 years of age	significantly more patients reported a reduction of pruritus intensity by ≥4 points on the Pruritus NRS scale compared to baseline after four weeks of therapy	not available
Cendakimab	IL-13	phase II trial (NCT04800315) [53]	adults	recently completed phase II clinical trial in AD, but no results available	not available
Eblasakimab	IL-13Rα1	proof-of-concept study [53]	adults	significant reduction of PP-NRS in comparison to placebo after eight weeks of therapy	not available
CM310	IL-4Rα	phase IIb trial (NCT04805411) [54]	adults	significantly lower pruritus intensity than in placebo, i.e., a reduction of ≥ 4 points in weekly mean daily PP-NRS compared to baseline—antipruritic effect noted in the second week and increased up to week 16	not available
Nemolizumab	IL-31 RA	phase II trial (NCT01986933) [56,57] phase IIb trial (NCT03100344) [58] phase III clinical trials (JapicCTI-173740 and JapicCTI-183894) [59]	NCT01986933 and NCT03100344: adults JapicCTI-173740 and JapicCTI-183894: over 13 years of age	confirmed reduction of pruritus intensity *NCT01986933* –dose-dependent antipruritic effect in the first week reported as a positive least-squares mean percentage change from baseline in VAS scale; antipruritic effects maintained or intensified up to week 64 *NCT03100344* –statistically significant reduction in the intensity of pruritus compared to placebo in the first week of therapy for all regimens *JapicCTI-173740* and *JapicCTI-183894* –after 68 week-long therapy a substantial decrease in the VAS scale reported in comparison to beginning of treatment	not available
Fezakinumab	IL-22	phase IIa clinical trial (NCT01941537) [60]	adults	no confirmed reduction of pruritus intensity	not available
Tezepelumab	TSLP	phase IIa clinical trial (NCT02525094) [62]	adults	significant difference in reduction of itch intensity based on Pruritus NRS scoring, but no statistically significant difference in the number of patients reaching EASI 50 at week 12 between tezepelumab groups and placebo	not available
Etokimab	IL-33	phase IIa proof-of- concept study (EudraCT 2016-002539-14) [26] phase IIb clinical study ATLAS (NCT03533751) [64]	adults	*EudraCT 2016-002539-14* significant reduction in pruritus intensity and incidence rate reported in the 5-D Itch Scale [26] *ATLAS* none of the groups reached primary outcome [64]	not available
Astegolimab	ST2 receptor	phase II study ZARNIE (NCT03747575) [65]	adults	no statistically significant changes after 16 weeks of therapy in any analyzed parameters (EASI, IGA, SCORAD, BSA, Pruritus NRS)	not available
Spesolimab	IL-36R	phase II study (NCT03747575) [66]	adults	no data available	not available
Ustekinumab	p40 IL-12/ IL-23	phase II study (NCT01806662) [71] phase II study (NCT01945086) [72]	adults	no satisfactory results in phase II placebo-controlled clinical trials [71,72]	effectiveness not confirmed [73]
Risankizumab	p19 IL-23	phase II clinical study (NCT03706040) [74]	over 14 years of age	unsatisfactory reduction of pruritus intensity at week 16 of therapy	not available
Secukinumab	IL-17A	phase II clinical trial (NCT02594098) [75]	adults	no clinically significant response to the treatment at week 16 of therapy	not available
MOR106	IL-17C	phase I and II clinical trials (NCT03568071, NCT03689829, NCT03689829, NCT03864627) [76]	adults	no satisfactory results in atopic dermatitis therapy with regard to skin changes or pruritus	not available
Rocatinlimab	OX40 receptor	phase IIb clinical trial (NCT03703102) [29]	adults	higher percentage of patients with a reduction of pruritus of ≥4 points in Pruritus NRS scale compared to placebo at week 16	not available
Amlitelimab	OX40L	phase IIa clinical study [77]	adults	57.9–62.5% of patients demonstrated a reduction of pruritus intensity of ≥4 points in Pruritus NRS scale	not available
Benralizumab	⍺-chain of the IL-5 receptor (IL-5R)	phase II clinical trial HILLIER (NCT04605094) [81]	over 12 years of age	no difference between placebo and study groups with regard to the number of patients achieving primary and secondary outcomes	not available
Mepolizumab	IL-5	phase II clinical study (NCT03055195) [82]	adults	research terminated due to no satisfactory clinical results	not available
Omalizumab	Cɛ3 IgE fragment	The Atopic Dermatitis Anti-IgE Pediatric Trial ADAPT (NCT02300701) [86] NCT00822783 [83] NCT01678092 [84]	ADAPT: children and adolescents (aged 4 to 19) NCT00822783: adults NCT01678092: children and adults	questionable in the treatment of atopic dermatitis [83,84]; some patients exhibited improvements in skin lesions and pruritus [85,86,87]	only case series available [85,87]
Ligelizumab	IgE	Eudra CT Number 2011-002112-84 [88]	adults	no satisfactory results in treating skin changes and reducing pruritus intensity	not available

## References

[1] Schuler C.F., Billi A.C., Maverakis E., Tsoi L.C., Gudjonsson J.E. (2023). Novel insights into atopic dermatitis. J. Allergy Clin. Immunol..

[2] Ramírez-Marín H.A., Silverberg J.I. (2022). Differences between pediatric and adult atopic dermatitis. Pediatr. Dermatol..

[3] Wu Y., Gu C., Wang S., Yin H., Qiu Z., Luo Y., Li Z., Wang C., Yao X., Li W. (2023). Serum biomarker-based endotypes of atopic dermatitis in China and prediction for efficacy of dupilumab. Br. J. Dermatol..

[4] Maintz L., Welchowski T., Herrmann N., Brauer J., Traidl-Hoffmann C., Havenith R., Müller S., Rhyner C., Dreher A., Schmid M. (2023). IL-13, periostin and dipeptidyl-peptidase-4 reveal endotype-phenotype associations in atopic dermatitis. Allergy.

[5] Bakker D., de Bruin-Weller M., Drylewicz J., van Wijk F., Thijs J. (2023). Biomarkers in atopic dermatitis. J. Allergy Clin. Immunol..

[6] Neri I., Galli E., Baiardini I., Picozza M., Rossi A.B., Matruglio P., Moretti D., Cipriani F. (2023). Implications of Atopic Dermatitis on the Quality of Life of 6-11 Years Old Children and Caregivers (PEDI-BURDEN). J. Asthma Allergy.

[7] Lundin S., Bergström A., Wahlgren C.F., Johansson E.K., Andersson N., Ballardini N., Jonsson M., Melén E., Kull I. (2022). Living with Atopic Dermatitis as a Young Adult in Relation to Health-related Quality of Life and Healthcare Contacts: A Population-based Study. Acta Derm. Venereol..

[8] Reynolds M., Gorelick J., Bruno M. (2020). Atopic Dermatitis: A Review of Current Diagnostic Criteria and a Proposed Update to Management. J. Drugs Dermatol..

[9] Yosipovitch G., Reaney M., Mastey V., Eckert L., Abbé A., Nelson L., Clark M., Williams N., Chen Z., Ardeleanu M. (2019). Peak Pruritus Numerical Rating Scale: Psychometric validation and responder definition for assessing itch in moderate-to-severe atopic dermatitis. Br. J. Dermatol..

[10] Reich A., Riepe C., Anastasiadou Z., Mędrek K., Augustin M., Szepietowski J.C., Ständer S. (2016). Itch Assessment with Visual Analogue Scale and Numerical Rating Scale: Determination of Minimal Clinically Important Difference in Chronic Itch. Acta Derm. Venereol..

[11] Kimball A.B., Naegeli A.N., Edson-Heredia E., Lin C.Y., Gaich C., Nikaï E., Wyrwich K., Yosipovitch G. (2016). Psychometric properties of the Itch Numeric Rating Scale in patients with moderate-to-severe plaque psoriasis. Br. J. Dermatol..

[12] Sroka-Tomaszewska J., Trzeciak M. (2021). Molecular Mechanisms of Atopic Dermatitis Pathogenesis. Int. J. Mol. Sci..

[13] Pontikas A., Antonatos C., Evangelou E., Vasilopoulos Y. (2023). Candidate Gene Association Studies in Atopic Dermatitis in Participants of European and Asian Ancestry: A Systematic Review and Meta-Analysis. Genes.

[14] Drislane C., Irvine A.D. (2020). The role of filaggrin in atopic dermatitis and allergic disease. Ann. Allergy Asthma Immunol..

[15] Akhtar S., Alsayed R.K.M.E., Ahmad F., AlHammadi A., Al-Khawaga S., AlHarami S.M.A.M., Alam M.A., Al Naama K.A.H.N., Buddenkotte J., Uddin S. (2023). Epigenetic control of inflammation in Atopic Dermatitis. Semin. Cell Dev. Biol..

[16] Fadadu R.P., Abuabara K., Balmes J.R., Hanifin J.M., Wei M.L. (2023). Air Pollution and Atopic Dermatitis, from Molecular Mechanisms to Population-Level Evidence: A Review. Int. J. Environ. Res. Public Health.

[17] Gu X., Jing D., Xiao Y., Zhou G., Yang S., Liu H., Chen X., Shen M. (2023). Association of air pollution and genetic risks with incidence of elderly-onset atopic dermatitis: A prospective cohort study. Ecotoxicol. Environ. Saf..

[18] Zhang S., Sumpter T.L., Kaplan D.H. (2022). Neuron-Mast Cell Cross-Talk in the Skin. J. Investig. Dermatol..

[19] Liu A.W., Gillis J.E., Sumpter T.L., Kaplan D.H. (2023). Neuroimmune interactions in atopic and allergic contact dermatitis. J. Allergy Clin. Immunol..

[20] Tominaga M., Takamori K. (2022). Peripheral itch sensitization in atopic dermatitis. Allergol. Int..

[21] Cevikbas F., Ward A., Firth C., Veverka K. (2023). Eblasakimab, a novel IL-13 receptor alpha 1 monoclonal antibody, blocks STAT6 phosphorylation with low dose in human volunteers. Clin. Immunol..

[22] Steinhoff M., Ahmad F., Pandey A., Datsi A., AlHammadi A., Al-Khawaga S., Al-Malki A., Meng J., Alam M., Buddenkotte J. (2022). Neuroimmune communication regulating pruritus in atopic dermatitis. J. Allergy Clin. Immunol..

[23] Yang B., Wilkie H., Das M., Timilshina M., Bainter W., Woods B., Daya M., Boorgula M.P., Mathias R.A., Lai P. (2023). The IL-4Rα Q576R polymorphism is associated with increased severity of atopic dermatitis and exaggerates allergic skin inflammation in mice. J. Allergy Clin. Immunol..

[24] Szegedi K., Lutter R., Res P.C., Bos J.D., Luiten R.M., Kezic S., Middelkamp-Hup M.A. (2015). Cytokine profiles in interstitial fluid from chronic atopic dermatitis skin. J. Eur. Acad. Dermatol. Venereol..

[25] Okragly A.J., Ryuzoji A., Wulur I., Daniels M., Van Horn R.D., Patel C.N., Benschop R.J. (2023). Binding, Neutralization and Internalization of the Interleukin-13 Antibody, Lebrikizumab. Dermatol. Ther..

[26] Chen Y.L., Gutowska-Owsiak D., Hardman C.S., Westmoreland M., MacKenzie T., Cifuentes L., Waithe D., Lloyd-Lavery A., Marquette A., Londei M. (2019). Proof-of-concept clinical trial of etokimab shows a key role for IL-33 in atopic dermatitis pathogenesis. Sci. Transl. Med..

[27] Trier A.M., Mack M.R., Fredman A., Tamari M., Ver Heul A.M., Zhao Y., Guo C.J., Avraham O., Ford Z.K., Oetjen L.K. (2022). IL-33 signaling in sensory neurons promotes dry skin itch. J. Allergy Clin. Immunol..

[28] Lopez D.V., Kongsbak-Wismann M. (2022). Role of IL-22 in homeostasis and diseases of the skin. APMIS.

[29] Guttman-Yassky E., Simpson E.L., Reich K., Kabashima K., Igawa K., Suzuki T., Mano H., Matsui T., Esfandiari E., Furue M. (2023). An anti-OX40 antibody to treat moderate-to-severe atopic dermatitis: A multicentre, double-blind, placebo-controlled phase 2b study. Lancet.

[30] Lou H., Lu J., Choi E.B., Oh M.H., Jeong M., Barmettler S., Zhu Z., Zheng T. (2017). Expression of IL-22 in the Skin Causes Th2-Biased Immunity, Epidermal Barrier Dysfunction, and Pruritus via Stimulating Epithelial Th2 Cytokines and the GRP Pathway. J. Immunol..

[31] Simpson E.L., Bieber T., Guttman-Yassky E., Beck L.A., Blauvelt A., Cork M.J., Silverberg J.I., Deleuran M., Kataoka Y., Lacour J.P. (2016). SOLO 1 and SOLO 2 Investigators. Two Phase 3 Trials of Dupilumab versus Placebo in Atopic Dermatitis. N. Engl. J. Med..

[32] Silverberg J.I., Yosipovitch G., Simpson E.L., Kim B.S., Wu J.J., Eckert L., Guillemin I., Chen Z., Ardeleanu M., Bansal A. (2020). Dupilumab treatment results in early and sustained improvements in itch in adolescents and adults with moderate to severe atopic dermatitis: Analysis of the randomized phase 3 studies SOLO 1 and SOLO 2, AD ADOL, and CHRONOS. J. Am. Acad. Dermatol..

[33] Alexis A.F., Rendon M., Silverberg J.I., Pariser D.M., Lockshin B., Griffiths C.E., Weisman J., Wollenberg A., Chen Z., Davis J.D. (2019). Efficacy of Dupilumab in Different Racial Subgroups of Adults with Moderate-to-Severe Atopic Dermatitis in Three Randomized, Placebo-Controlled Phase 3 Trials. J. Drugs Dermatol..

[34] de Bruin-Weller M., Thaçi D., Smith C.H., Reich K., Cork M.J., Radin A., Zhang Q., Akinlade B., Gadkari A., Eckert L. (2018). Dupilumab with concomitant topical corticosteroid treatment in adults with atopic dermatitis with an inadequate response or intolerance to ciclosporin A or when this treatment is medically inadvisable: A placebo-controlled, randomized phase III clinical trial (LIBERTY AD CAFÉ). Br. J. Dermatol..

[35] Beck L.A., Thaçi D., Deleuran M., Blauvelt A., Bissonnette R., de Bruin-Weller M., Hide M., Sher L., Hussain I., Chen Z. (2020). Dupilumab Provides Favorable Safety and Sustained Efficacy for up to 3 Years in an Open-Label Study of Adults with Moderate-to-Severe Atopic Dermatitis. Am. J. Clin. Dermatol..

[36] Beck L.A., Deleuran M., Bissonnette R., de Bruin-Weller M., Galus R., Nakahara T., Seo S.J., Khokhar F.A., Vakil J., Xiao J. (2022). Dupilumab Provides Acceptable Safety and Sustained Efficacy for up to 4 Years in an Open-Label Study of Adults with Moderate-to-Severe Atopic Dermatitis. Am. J. Clin. Dermatol..

[37] Silverberg J.I., Rubini N.P.M., Pires M.C., Rossi A.B., Zhang A., Chen Z., Levit N.A., Chao J., Shumel B., Bégo-Le Bagousse G. (2022). Dupilumab Treatment Reduces Hospitalizations in Adults With Moderate-to-Severe Atopic Dermatitis. J. Allergy Clin. Immunol. Pract..

[38] Ariëns L.F.M., van der Schaft J., Spekhorst L.S., Bakker D.S., Romeijn G.L.E., Kouwenhoven T.A., Kamsteeg M., Voorberg A.N., Oosting A.J., de Ridder I. (2021). Dupilumab shows long-term effectiveness in a large cohort of treatment-refractory atopic dermatitis patients in daily practice: 52-Week results from the Dutch BioDay registry. J. Am. Acad. Dermatol..

[39] Salman A., Apti Sengun Ö., Aktas M., Taşkapan O. (2022). Real-life effectiveness and safety of dupilumab in adult patients with moderate-to-severe atopic dermatitis. Dermatol. Ther..

[40] Fargnoli M.C., Esposito M., Ferrucci S., Girolomoni G., Offidani A., Patrizi A., Peris K., Costanzo A., Malara G., Pellacani G. (2021). Dupilumab Italian National Access Program (Dup-INAP group). Real-life experience on effectiveness and safety of dupilumab in adult patients with moderate-to-severe atopic dermatitis. J. Dermatolog. Treat..

[41] Olesen C.M., Holm J.G., Nørreslet L.B., Serup J.V., Thomsen S.F., Agner T. (2019). Treatment of atopic dermatitis with dupilumab: Experience from a tertiary referral centre. J. Eur. Acad. Dermatol. Venereol..

[42] Faiz S., Giovannelli J., Podevin C., Jachiet M., Bouaziz J.D., Reguiai Z., Nosbaum A., Lasek A., Ferrier le Bouedec M.C., Du Thanh A. (2019). Effectiveness and safety of dupilumab for the treatment of atopic dermatitis in a real-life French multicenter adult cohort. J. Am. Acad. Dermatol..

[43] Simpson E.L., Wollenberg A., Soong W., Steffensen L.A., Kurbasic A., Schneider S., Zoidis J., Silverberg J.I. (2022). Patient-oriented measures for phase 3 studies of tralokinumab for the treatment of atopic dermatitis (ECZTRA 1, 2, and 3). Ann. Allergy Asthma Immunol..

[44] Gutermuth J., Pink A.E., Worm M., Soldbro L., Bjerregård Øland C., Weidinger S. (2022). Tralokinumab plus topical corticosteroids in adults with severe atopic dermatitis and inadequate response to or intolerance of ciclosporin A: A placebo-controlled, randomized, phase III clinical trial (ECZTRA 7). Br. J. Dermatol..

[45] Simpson E.L., Merola J.F., Silverberg J.I., Reich K., Warren R.B., Staumont-Sallé D., Girolomoni G., Papp K., de Bruin-Weller M., Thyssen J.P. (2022). Safety of tralokinumab in adult patients with moderate-to-severe atopic dermatitis: Pooled analysis of five randomized, double-blind, placebo-controlled phase II and phase III trials. Br. J. Dermatol..

[46] Blauvelt A., Langley R.G., Lacour J.P., Toth D., Laquer V., Beissert S., Wollenberg A., Herranz P., Pink A.E., Peris K. (2022). Long-term 2-year safety and efficacy of tralokinumab in adults with moderate-to-severe atopic dermatitis: Interim analysis of the ECZTEND open-label extension trial. J. Am. Acad. Dermatol..

[47] De Greef A., Ghislain P.D., Bulinckx A., Coster A., de Halleux C., Damsin T., Jacobs M.C., Suys E., Zoghaib S., Baeck M. (2023). Real-Life Experience of Tralokinumab for the Treatment of Adult Patients with Severe Atopic Dermatitis: A Multicentric Prospective Study. Clin. Drug. Investig..

[48] Pereyra-Rodríguez J.J., Herranz P., Ruiz-Villaverde R., Elosua-González M., Galán-Gutiérrez M., Figueras-Nart I., Miquel J., de la Cueva P., Serra-Baldrich E., Munera-Campos M. (2023). Treatment of severe atopic dermatitis with tralokinumab in clinical practice: Short-term effectiveness and safety results. Clin. Exp. Dermatol..

[49] Silverberg J.I., Guttman-Yassky E., Thaçi D., Irvine A.D., Stein Gold L., Blauvelt A., Simpson E.L., Chu C.Y., Liu Z., Gontijo Lima R. (2023). ADvocate1 and ADvocate2 Investigators. Two Phase 3 Trials of Lebrikizumab for Moderate-to-Severe Atopic Dermatitis. N. Engl. J. Med..

[50] Blauvelt A., Thyssen J.P., Guttman-Yassky E., Bieber T., Serra-Baldrich E., Simpson E., Rosmarin D., Elmaraghy H., Meskimen E., Natalie C.R. (2023). Efficacy and safety of lebrikizumab in moderate-to-severe atopic dermatitis: 52-week results of two randomized double-blinded placebo-controlled phase III trials. Br. J. Dermatol..

[51] Stein Gold L., Thaçi D., Thyssen J.P., Gooderham M., Laquer V., Moore A., Natalie C.R., Zhao F., Meskimen E., Elmaraghy H. (2023). Safety of Lebrikizumab in Adults and Adolescents with Moderate-to-Severe Atopic Dermatitis: An Integrated Analysis of Eight Clinical Trials. Am. J. Clin. Dermatol..

[52] Hirano I., Rothenberg M.E., Zhang S., de Oliveira C., Charriez C.M., Coyne K.S., Bacci E.D., Dellon E.S. (2023). Dysphagia Days as an Assessment of Clinical Treatment Outcome in Eosinophilic Esophagitis. Am. J. Gastroenterol..

[53] Lytvyn Y., Gooderham M. (2023). Targeting Interleukin 13 for the Treatment of Atopic Dermatitis. Pharmaceutics.

[54] Zhao Y., Zhang J., Yang B., Li J., Ding Y., Wu L., Zhang L., Wang J., Zhu X., Zhang F. (2023). Efficacy and safety of CM310 in moderate-to-severe atopic dermatitis: A multicenter, randomized, double-blind, placebo-controlled phase 2b trial. Chin. Med. J..

[55] ClinicalTrials.gov. https://clinicaltrials.gov/.

[56] Kabashima K., Furue M., Hanifin J.M., Pulka G., Wollenberg A., Galus R., Etoh T., Mihara R., Nakano M., Ruzicka T. (2018). Nemolizumab in patients with moderate-to-severe atopic dermatitis: Randomized, phase II, long-term extension study. J. Allergy Clin. Immunol..

[57] Ruzicka T., Hanifin J.M., Furue M., Pulka G., Mlynarczyk I., Wollenberg A., Galus R., Etoh T., Mihara R., Yoshida H. (2017). XCIMA Study Group. Anti-Interleukin-31 Receptor A Antibody for Atopic Dermatitis. N. Engl. J. Med..

[58] Silverberg J.I., Pinter A., Pulka G., Poulin Y., Bouaziz J.D., Wollenberg A., Murrell D.F., Alexis A., Lindsey L., Ahmad F. (2020). Phase 2B randomized study of nemolizumab in adults with moderate-to-severe atopic dermatitis and severe pruritus. J. Allergy Clin. Immunol..

[59] Kabashima K., Matsumura T., Komazaki H., Kawashima M. (2022). Nemolizumab JP01 andJP02 Study Group. Nemolizumab plus topical agents in patients with atopic dermatitis (AD) and moderate-to-severe pruritus provide improvement in pruritus and signs of AD for up to 68 weeks: Results from two phase III, long-term studies. Br. J. Dermatol..

[60] Guttman-Yassky E., Brunner P.M., Neumann A.U., Khattri S., Pavel A.B., Malik K., Singer G.K., Baum D., Gilleaudeau P., Sullivan-Whalen M. (2018). Efficacy and safety of fezakinumab (an IL-22 monoclonal antibody) in adults with moderate-to-severe atopic dermatitis inadequately controlled by conventional treatments: A randomized, double-blind, phase 2a trial. J. Am. Acad. Dermatol..

[61] Brunner P.M., Pavel A.B., Khattri S., Leonard A., Malik K., Rose S., Jim On S., Vekaria A.S., Traidl-Hoffmann C., Singer G.K. (2019). Baseline IL-22 expression in patients with atopic dermatitis stratifies tissue responses to fezakinumab. J. Allergy Clin. Immunol..

[62] Simpson E.L., Parnes J.R., She D., Crouch S., Rees W., Mo M., van der Merwe R. (2019). Tezepelumab, an anti-thymic stromal lymphopoietin monoclonal antibody, in the treatment of moderate to severe atopic dermatitis: A randomized phase 2a clinical trial. J. Am. Acad. Dermatol..

[63] Parnes J.R., Sullivan J.T., Chen L., Dias C. (2019). Pharmacokinetics, Safety, and Tolerability of Tezepelumab (AMG 157) in Healthy and Atopic Dermatitis Adult Subjects. Clin. Pharmacol. Ther..

[64] Uppal S.K., Kearns D.G., Chat V.S., Han G., Wu J.J. (2022). Review and analysis of biologic therapies currently in phase II and phase III clinical trials for atopic dermatitis. J. Dermatolog. Treat..

[65] Maurer M., Cheung D.S., Theess W., Yang X., Dolton M., Guttman A., Choy D.F., Dash A., Grimbaldeston M.A., Soong W. (2022). Phase 2 randomized clinical trial of astegolimab in patients with moderate to severe atopic dermatitis. J. Allergy Clin. Immunol..

[66] Bissonnette R., Abramovits W., Saint-Cyr Proulx É., Lee P., Guttman-Yassky E., Zovko E., Sigmund R., Willcox J., Bieber T. (2023). Spesolimab, an anti-interleukin-36 receptor antibody, in patients with moderate-to-severe atopic dermatitis: Results from a multicentre, randomized, double-blind, placebo-controlled, phase IIa study. J. Eur. Acad. Dermatol. Venereol..

[67] Agusti-Mejias A., Messeguer F., García R., Febrer I. (2013). Severe refractory atopic dermatitis in an adolescent patient successfully treated with ustekinumab. Ann. Dermatol..

[68] Fernández-Antón Martínez M.C., Alfageme Roldán F., Ciudad Blanco C., Suárez Fernández R. (2014). Ustekinumab in the treatment of severe atopic dermatitis: A preliminary report of our experience with 4 patients. Actas Dermosifiliogr..

[69] Wlodek C., Hewitt H., Kennedy C.T. (2016). Use of ustekinumab for severe refractory atopic dermatitis in a young teenager. Clin. Exp. Dermatol..

[70] Puya R., Alvarez-López M., Velez A., Casas Asuncion E., Moreno J.C. (2012). Treatment of severe refractory adult atopic dermatitis with ustekinumab. Int. J. Dermatol..

[71] Khattri S., Brunner P.M., Garcet S., Finney R., Cohen S.R., Oliva M., Dutt R., Fuentes-Duculan J., Zheng X., Li X. (2017). Efficacy and safety of ustekinumab treatment in adults with moderate-to-severe atopic dermatitis. Exp. Dermatol..

[72] Saeki H., Kabashima K., Tokura Y., Murata Y., Shiraishi A., Tamamura R., Randazzo B., Imanaka K. (2017). Efficacy and safety of ustekinumab in Japanese patients with severe atopic dermatitis: A randomized, double-blind, placebo-controlled, phase II study. Br. J. Dermatol..

[73] Husein-ElAhmed H., Steinhoff M. (2022). Effectiveness of ustekinumab in patients with atopic dermatitis: Analysis of real-world evidence. J. Dermatol. Treat..

[74] Tyring S.K., Rich P., Tada Y., Beeck S., Messina I., Liu J., Huang X., Shumack S. (2023). Risankizumab in Patients with Moderate-to-Severe Atopic Dermatitis: A Phase 2, Randomized, Double-Blind, Placebo-Controlled Study. Dermatol. Ther..

[75] Ungar B., Pavel A.B., Li R., Kimmel G., Nia J., Hashim P., Kim H.J., Chima M., Vekaria A.S., Estrada Y. (2021). Phase 2 randomized, double-blind study of IL-17 targeting with secukinumab in atopic dermatitis. J. Allergy Clin. Immunol..

[76] Thaçi D., Singh D., Lee M., Timmis H., Jacobs D., Passier P., Rohrer S., Beetens J., Phung D., Sondag E. (2022). Phase 1 and 2 Randomized Clinical Studies Determine Lack of Efficacy for Anti-IL-17C Antibody MOR106 in Moderate-Severe Atopic Dermatitis. J. Clin. Med..

[77] Lé A.M., Torres T. (2022). OX40-OX40L Inhibition for the Treatment of Atopic Dermatitis-Focus on Rocatinlimab and Amlitelimab. Pharmaceutics.

[78] Iriki H., Takahashi H., Amagai M. (2023). Diverse Role of OX40 on T Cells as a Therapeutic Target for Skin Diseases. J. Investig. Dermatol..

[79] Saghari M., Gal P., Gilbert S., Yateman M., Porter-Brown B., Brennan N., Quaratino S., Wilson R., Grievink H.W., Klaassen E.S. (2022). OX40L Inhibition Suppresses KLH-driven Immune Responses in Healthy Volunteers: A Randomized Controlled Trial Demonstrating Proof-of-Pharmacology for KY1005. Clin. Pharmacol. Ther..

[80] Pham D.N. (2021). Spontaneous resolution of atopic dermatitis incidental to participation in benralizumab clinical trial for severe, uncontrolled asthma: A case report. J. Med. Case Rep..

[81] Guttman-Yassky E., Bahadori L., Brooks L., Clark K.L., Grindebacke H., Ho C.N., Katial R., Pham T.H., Walton C., Datto C.J. (2023). Lack of effect of benralizumab on signs and symptoms of moderate-to-severe atopic dermatitis: Results from the phase 2 randomized, double-blind, placebo-controlled HILLIER trial. J. Eur. Acad. Dermatol. Venereol..

[82] Kang E.G., Narayana P.K., Pouliquen I.J., Lopez M.C., Ferreira-Cornwell M.C., Getsy J.A. (2020). Efficacy and safety of mepolizumab administered subcutaneously for moderate to severe atopic dermatitis. Allergy.

[83] Heil P.M., Maurer D., Klein B., Hultsch T., Stingl G. (2010). Omalizumab therapy in atopic dermatitis: Depletion of IgE does not improve the clinical course—A randomized, placebo-controlled and double blind pilot study. J. Dtsch. Dermatol. Ges..

[84] Iyengar S.R., Hoyte E.G., Loza A., Bonaccorso S., Chiang D., Umetsu D.T., Nadeau K.C. (2013). Immunologic effects of omalizumab in children with severe refractory atopic dermatitis: A randomized, placebo-controlled clinical trial. Int. Arch. Allergy Immunol..

[85] Fernández-Antón Martínez M.C., Leis-Dosil V., Alfageme-Roldán F., Paravisini A., Sánchez-Ramón S., Suárez Fernández R. (2012). Omalizumab for the treatment of atopic dermatitis. Actas Dermosifiliogr..

[86] Chan S., Cornelius V., Cro S., Harper J.I., Lack G. (2020). Treatment Effect of Omalizumab on Severe Pediatric Atopic Dermatitis: The ADAPT Randomized Clinical Trial. JAMA Pediatr..

[87] Holm J.G., Agner T., Sand C., Thomsen S.F. (2017). Omalizumab for atopic dermatitis: Case series and a systematic review of the literature. Int. J. Dermatol..

[88] Bangert C., Loesche C., Skvara H., Fölster-Holst R., Lacour J.P., Jones J., Burnett P., Novak N., Stingl G. (2023). IgE Depletion with Ligelizumab Does Not Significantly Improve Clinical Symptoms in Patients with Modlerate-to-Severe Atopic Dermatitis. J. Investig. Dermatol..

